# Ecto-ADP-ribosyltransferase ARTC2.1 functionally modulates FcγR1 and FcγR2B on murine microglia

**DOI:** 10.1038/s41598-017-16613-w

**Published:** 2017-11-28

**Authors:** Björn Rissiek, Stephan Menzel, Mario Leutert, Maike Cordes, Sarah Behr, Larissa Jank, Peter Ludewig, Mathias Gelderblom, Anne Rissiek, Sahil Adriouch, Friedrich Haag, Michael O. Hottiger, Friedrich Koch-Nolte, Tim Magnus

**Affiliations:** 1 Department of Neurology, University Medical Center Hamburg-Eppendorf, Hamburg, Germany; 2 Institute of Immunology, University Medical Center Hamburg-Eppendorf, Hamburg, Germany; 3 Department of Molecular Mechanisms of Disease, University of Zurich, Zurich, Switzerland; 4 Normandie Univ, UNIROUEN, INSERM, U1234, Institute for Research and Innovation in Biomedicine (IRIB), Rouen, France

## Abstract

Mammalian ecto-ADP-ribosyltransferases (ecto-ARTs or also ARTCs) catalyze the ADP-ribosylation of cell surface proteins using extracellular nicotinamide adenine dinucleotide (NAD^+^) as substrate. By this post-translational protein modification, ecto-ARTs modulate the function of various target proteins. A functional role of ARTC2 has been demonstrated for peripheral immune cells such as T cells and macrophages. Yet, little is known about the role of ecto-ARTs in the central nervous system and on microglia. Here, we identified ARTC2.1 as the major ecto-ART expressed on murine microglia. ARTC2.1 expression was strongly upregulated on microglia upon co-stimulation with LPS and an ERK1/2 inhibitor or upon IFNβ stimulation. We identified several target proteins modified by ARTC2.1 on microglia with a recently developed mass spectrometry approach, including two receptors for immunoglobulin G (IgG), FcγR1 and FcγR2B. Both proteins were verified as targets of ARTC2.1 *in vitro* using a radiolabeling assay with ^32^P-NAD^+^ as substrate. Moreover, ADP-ribosylation of both targets strongly inhibited their capacity to bind IgG. In concordance, ARTC2.1 induction in WT microglia and subsequent cell surface ADP-ribosylation significantly reduced the phagocytosis of IgG-coated latex beads, which was unimpaired in NAD^+^/DTT treated microglia from ARTC2.1^−/−^ mice. Hence, induction of ARTC2.1 expression under inflammatory conditions, and subsequent ADP-ribosylation of cell surface target proteins could represent a hitherto unnoticed mechanism to regulate the immune response of murine microglia.

## Introduction

Mammalian ecto-ADP-ribosyltransferases (ARTs) are cell surface enzymes that catalyze the covalent transfer of the ADP-ribose moiety from nicotinamide adenine dinucleotide (NAD^+^) to arginine residues on their target proteins^1^. Owing to their structural relation to clostridial toxins C2 and C3, mammalian ecto-ARTs are abbreviated “ARTCs”, whereas intracellular ARTs structurally related to diphtheria toxin are abbreviated “ARTDs” (formerly poly-ADP-ribosyltransferases (PARPs))^2^.

The murine ARTC family comprises 6 members, ARTC1-5 including two isoforms of ARTC2 (ARTC2.1 and ARTC2.2) that are encoded by two closely linked genes (*Art2a* and *Art2b)* ^3^. ARTC2.1 and ARTC2.2 display approximately 78% sequence identity. Interestingly, ARTC2.1 contains an extra allosteric disulfide bond and is active only under mild reducing conditions^4^. *Art2a* and *Art2b* are known to be differentially expressed among common laboratory mouse strains. While BALB/c mice functionally express both genes, a nonsense mutation in *Art2a* results in the absence of the ARTC2.1 enzyme in the C57BL/6 strain and a deletion of the *Art2b* gene results in absence of the ARTC2.2 enzyme in the NZW strain^5–7^. Both ARTC2 isoforms are prominently expressed on immune cells. While T cells predominantly express *Art2b* and, to a lower extent, *Art2a*, antigen presenting cells such as macrophages and dendritic cells only express *Art2a*. Interestingly, macrophage cell surface expression of ARTC2.1 can be strongly increased by priming the cells with proinflammatory mediators including agonists for Toll-like receptors (TLRs) and type I and type II interferons, suggesting that ARTC2.1 regulates cell responses during inflammatory conditions^8^.

Several ARTC2 target proteins have been identified at the surface of immune cells and the functional consequences of their ADP-ribosylation have been investigated. In case of the ligand-gated ion channel P2X7, ADP-ribosylation at arginine position 125 (Arg125) leads to its activation by providing a covalently attached ADP-ribose ligand in the vicinity of the ligand-binding pocket^9–11^. For several other ARTC2 targets, including LFA-1, CD8a, and CD25, ADP-ribosylation has been shown to inhibit binding to their natural ligands^12–15^. Hence, ADP-ribosylation of target proteins at the surface of immune cells by ART2 ecto-enzymes constitutes an important post-translational mechanism to regulate cell surface protein function and, thereby, immune cell functions.

Microglia are local innate immune cells of the central nervous system that exhibit similar properties as peripheral macrophages^16^. They can phagocytose invading pathogens or necrotic cells following ischemia, and they respond to pathogenic insults with the release of proinflammatory cytokines^17^. Even though the role of microglia in brain inflammation has been extensively studied, little is known about the expression and function of ecto-ARTs on these cells. The aims of this study were to characterize the expression and the activity of ARTC2 on microglia, to identify potential target proteins of ARTC2, and to study the functional consequences of their ADP-ribosylation. We found that ARTC2.1 expression on microglia is upregulated under inflammatory conditions and, using a novel mass spectrometry approach^18^, we identified FcγR1 and FcγR2B as prominent targets that are functionally inhibited by ADP-ribosylation.

## Results

### Ecto-ADP-ribosyltransferase activity is detectable on a small fraction of microglia from mixed glial cell cultures

**Figure 1:**
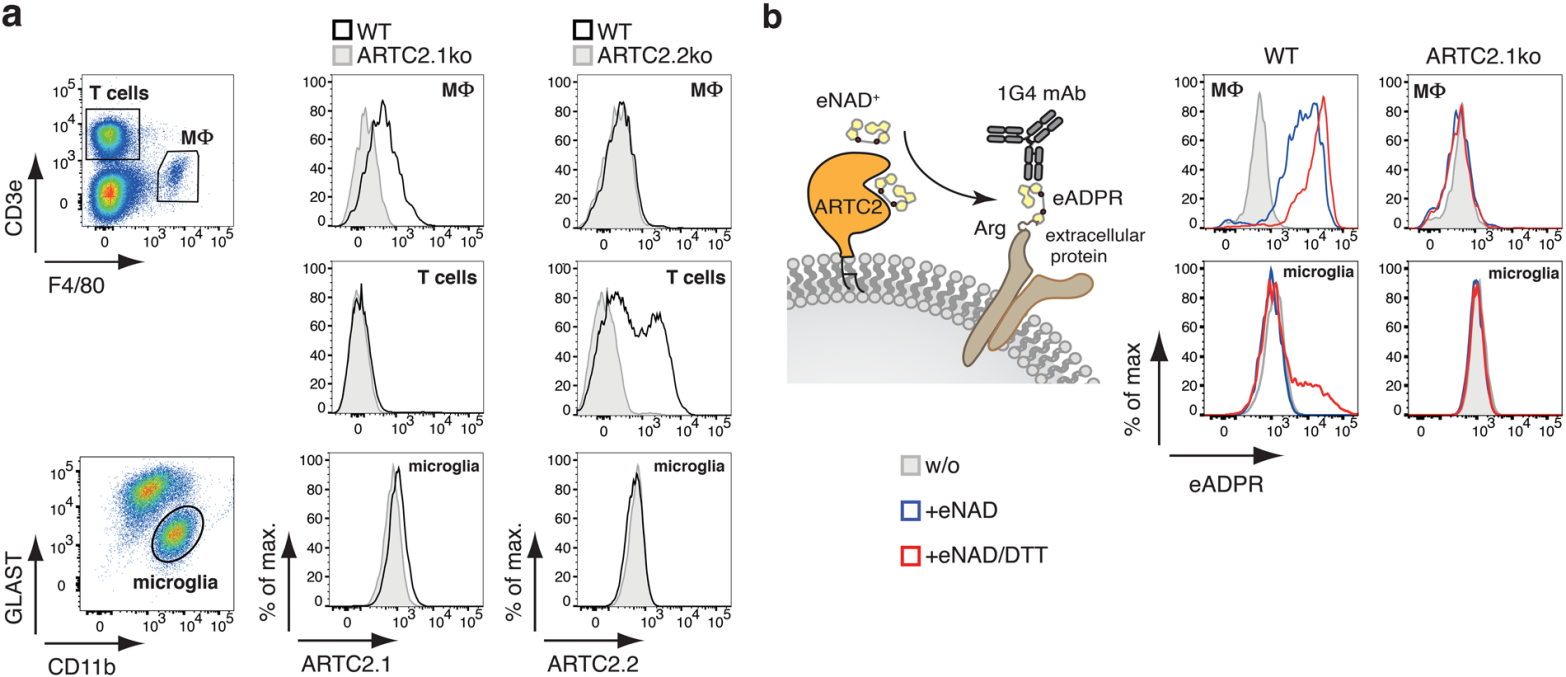
Ecto-ART expression and activity on microglia from mixed glial cell cultures. (**a**) Reactivity of fluorochrome-conjugated ARTC2.1 and ARTC2.2-specific mAbs was analyzed by flow cytometry of BALB/c WT spleen CD3^+^ T cells, F4/80^+^ macrophages (MΦ). or CD11b^+^GLAST^−^ microglia from BALB/c WT mixed glial cell cultures. BALB/c ARTC2.1^−/−^ and ARTC2.2^−/−^ cells were used as negative controls. (**b**) Etheno-NAD (eNAD^+^) can be used as surrogate substrate for ARTC2, which etheno-ADP-ribosylates cell surface proteins. This can be detected by use of etheno-adenosine-specific mAb 1G4. Ecto-ART activity was analyzed by measuring etheno-ADP-ribosylation of cell surface proteins on BALB/c WT and ARTC2.1^−/−^ spleen MΦ (upper panel) and on microglia from mixed glial cell cultures (lower panel) after incubating cells with 100 µM eNAD^+^ (blue), with 100 µM eNAD^+^ and 2 mM DTT (red) or without additions (grey). Etheno-ADP-ribosylated proteins were detected with mAb 1G4. Data are representative of 3 independent experiments.

To monitor expression of ARTC2 isoforms on the cell surface of microglia we used monoclonal antibodies (mAbs) that specifically detect ARTC2.1 (clone R18-A136) or ARTC2.2 (clone NIKA-A102)^7,19.^ With these we analyzed immune cells from BALB/c WT mice or ARTC2.1- and ARTC2.2-deficient BALB/c mice as respective negative controls. First, we confirmed the specificity of both ARTC2-specific mAbs on spleen F4/80^+^ macrophages, known to predominantly express ARTC2.1 and on spleen CD3e^+^ T cells, known to express high levels of ARTC2.2 (Fig. 1a). Indeed, by using ARTC2.1- and ARTC2.2-specific mAbs in flow cytometry analyses we confirmed the expression of ARTC2.1 on macrophages of WT but not ARTC2.1^−/−^ mice and the expression of ARTC2.2 on T cells of WT but not ARTC2.2^−/−^ mice. We then used the same isoform-specific mAbs to explore the expression of ARTC2.1/ARTC2.2 on CD11b^+^GLAST^−^ microglia obtained from mixed glial cell cultures. Using flow cytometry, we detected little if any ARTC2.1 expression on WT microglia compared to ARTC2.1^−/−^ microglia, while the ARTC2.2 expression was virtually absent on both, WT and ARTC2.2^−/−^ microglia (Fig. 1a).

We next used a more sensitive approach to detect the presence of ADP-ribosyltransferases (ART) enzymatic cell surface activity. Incubation of cells with the ARTC2 surrogate substrate etheno-nicotinamide adenine dinucleotide (eNAD^+^) induces the etheno-ADP-ribosylation (eADPR) of cell surface proteins catalyzed by ARTC2. The mAb 1G4 was then used to detect the eADPR cell surface proteins using flow cytometry^20^ (Fig. 1b). We first performed this assay using F4/80^+^ splenic macrophages. As expected, F4/80^+^ macrophages from WT mice exhibited a robust cell surface ART enzymatic activity that was further increased in the presence of the reducing agent dithiothreitol (DTT), a known enhancer of ARTC2.1 enzymatic activity^21^. In contrast, there was no detectable ART activity on macrophages of ARTC2.1^−/−^ mice (Fig. 1b). We next used the same methodology to monitor the enzymatic ART activity on microglia from mixed glial cell cultures. We observed that only a small fraction of microglia from WT mice exhibited cell surface ART activity in the presence of eNAD^+^/DTT (Fig. 1b). This enzymatic activity was virtually absent on microglia derived from ARTC2.1^−/−^ mice under the tested conditions. Hence, these results suggest a low, but enzymatically detectable, expression of ARTC2.1 at the surface of a subset of microglia.

### ARTC2.1 expression can be induced in microglia by co-stimulation with LPS and U0126

**Figure 2:**
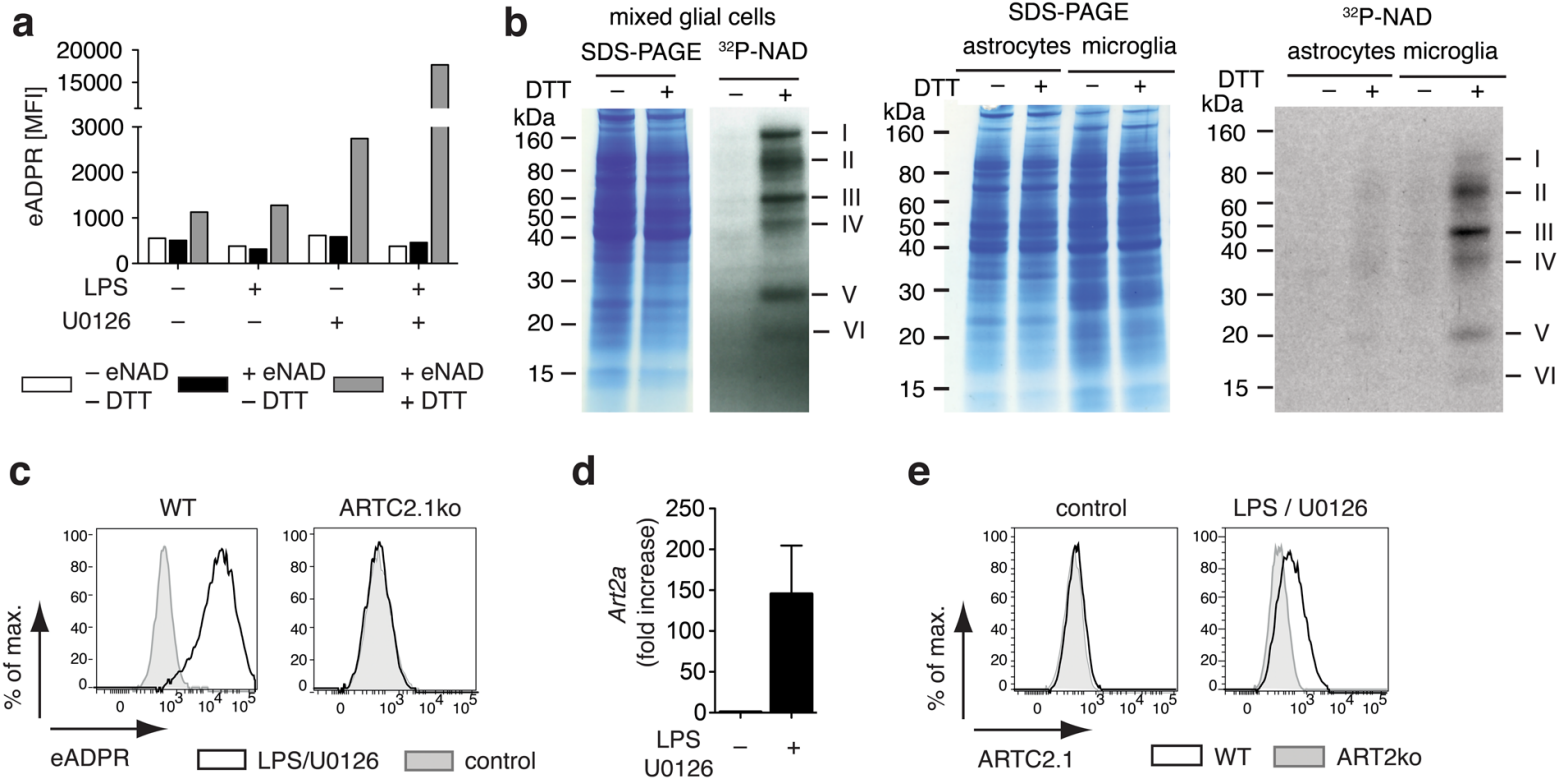
ARTC2.1 is upregulated on microglia upon stimulation with LPS and U0126. (**a**) Mixed glial cells were stimulated with or without LPS (0.1 µg/ml), U0126 (10 µM) or both for 24 h, followed by incubation with eNAD^+^ (50 µM) and DTT (2.5 mM) for 15 min. Etheno-ADP-ribosylated cell surface proteins were detected with mAb 1G4 (displayed as mean fluorescence intensity, MFI). (**b**) Mixed glial cell cultures were stimulated with LPS (0.1 µg/ml) and U0126 (10 µM) for 24 h. Total cells (left) or FACS sorted astrocytes and microglia (right) were then incubated for 15 min with radioactive ^32^P-NAD^+^ (1 µM) in the presence or absence of DTT (2.5 mM). Cells were lysed, proteins were size fractioned by SDS PAGE and subjected to autoradiography. (**c**) Mixed glial cells from BALB/c WT and ARTC2.1^−/−^ mice were stimulated with LPS (0.1 µg/ml) and U0126 (10 µM) for 24 h, and then incubated with eNAD^+^ (50 µM) and DTT (2.5 mM) for 15 min. Etheno-ADP-ribosylated surface proteins were detected with mAb 1G4. (**d**) mRNA levels of *Art2a* from FACS sorted microglia (n = 5 individual experiments) of unstimulated or LPS/U0126 stimulated mixed glial cell cultures were determined by quantitative real-time PCR. (**e**) Surface expression of ARTC2.1 by microglia of LPS/U0126 stimulated or control mixed glial cell cultures of BALB/c WT or ARTC2^−/−^ mice was analyzed by flow cytometry as in Fig. 1c. Data are representative of 2–3 independent experiments.

It has been described that macrophage stimulation with lipopolysaccharide (LPS) increases the expression of ARTC2.1 which can be further amplified by the ERK1/2 inhibitor U0126 (Sigma)^8,22^. Therefore, it is conceivable that ARTC2.1 expression on microglia can be enhanced by similar mechanisms. We thus stimulated mixed glial cells with LPS in the presence of U0126 for 24 h, and then evaluated the ART enzymatic activity present at the cell surface by flow cytometry. LPS treatment alone did not increase cell surface ART activity on microglia compared to untreated controls and U0126 alone only led to a slight increase of enzymatic activity on microglia. However, combined stimulation with LPS and U0126 resulted in a robust increase in cell surface ART activity on microglia (Fig. 2a). To further validate this finding, we incubated LPS/U0126 treated mixed glial cells with radioactive ^32^P-NAD^+^ in the presence or absence of DTT, lysed the cells and analyzed the solubilized proteins for incorporated radioactivity by SDS-PAGE autoradiography. We observed multiple prominent bands (I–VI) when cells were incubated with ^32^P-NAD^+^ in the presence of DTT, suggesting ADP-ribosylation of multiple target proteins (Fig. 2b). Further, we separated microglia and astrocytes from LPS/U0126 stimulated mixed glial cell cultures by FACS sorting, before incubation with radioactive ^32^P-NAD^+^/DTT and analysis of proteins by SDS-PAGE autoradiography. We observed a similar pattern of ADP-ribosylated proteins for microglia as observed for mixed glial cells, whereas astrocytes barely showed any ADP-ribosylation of cell surface proteins, indicating that microglia are the main source of cell surface ART activity in the mixed glial cell culture (Fig. 2b). As the enzymatic activity of ARTC2.1, in contrast to that of ARTC2.2, is strongly enhanced under reducing conditions, the absence of detectable ADP-ribosylated proteins in samples that were not treated with DTT suggested that ARTC2.1 was the main ecto-ART expressed by microglia. To further test this hypothesis, we repeated the experiment with mixed glial cells derived from ARTC2.1^−/−^ mice. The results show that microglia from ARTC2.1^−/−^ mice did not exhibit any detectable cell surface ART activity after LPS/U0126 stimulation(Fig. 2c).

We next investigated the upregulation of ARTC2.1 in microglia upon LPS/U0126 treatment. Quantification of *Art2a* mRNA by qRT-PCR analyses of FACS sorted microglia revealed a more than 100-fold higher level of *Art2a* mRNA in cells treated with LPS/U0126 versus untreated control cells (Fig. 2d). Using the ARTC2.1-specific mAb R18-A136 we confirmed the enhanced cell surface expression of ARTC2.1 on microglia after LPS/U0126 treatment (Fig. 2e). Taken together, the results show that ARTC2.1 on microglia is strongly upregulated by LPS/U0126 treatment, enabling ADP-ribosylation of multiple target proteins on microglia in the presence of the ARTC2.1 substrate NAD^+^.

### ARTC2.1 expression in microglia can be induced by IFNβ stimulation

**Figure 3:**
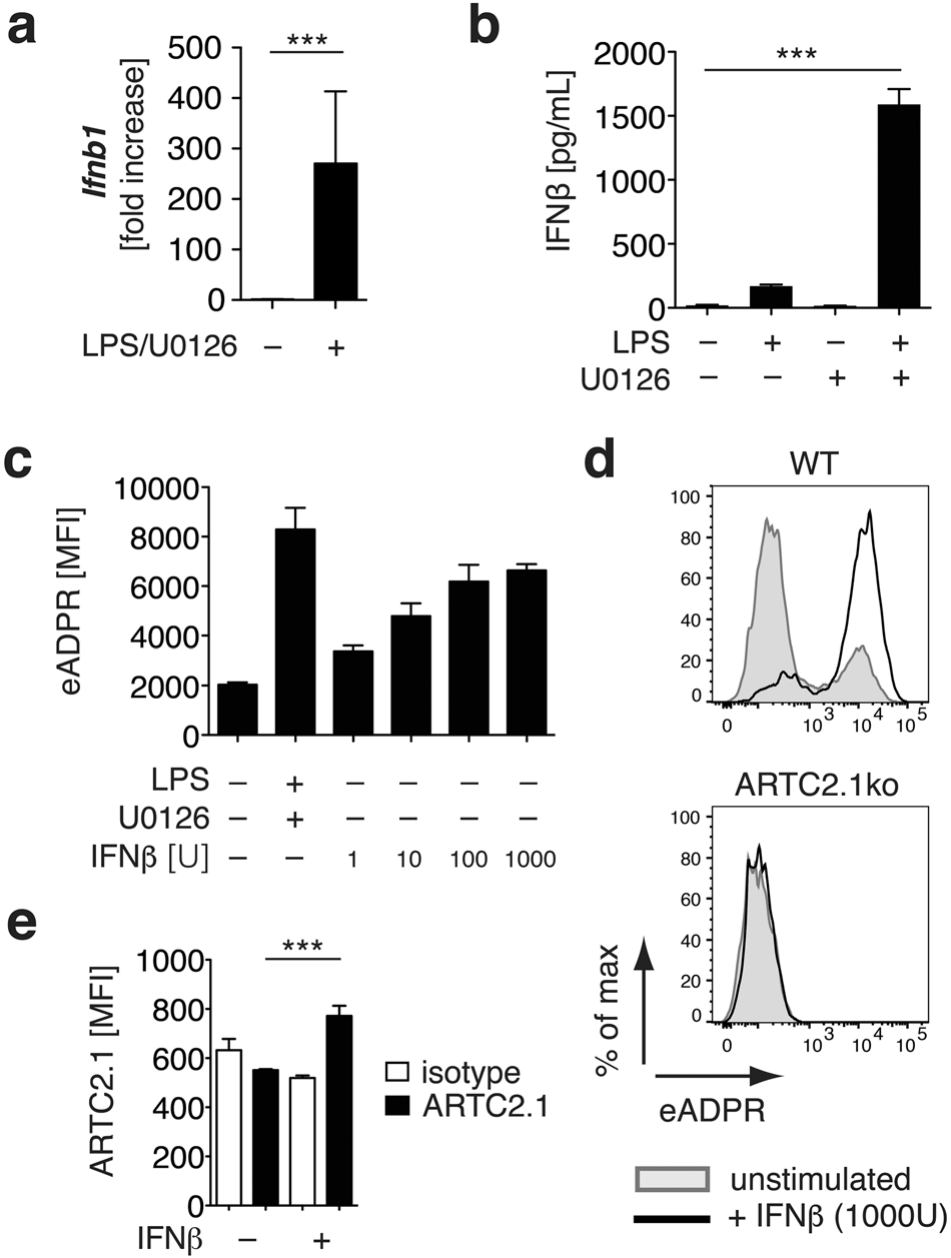
ARTC2.1 is upregulated on microglia upon stimulation with IFNβ. (**a)** mRNA levels of *Ifnb1* from FACS sorted microglia (n = 5 individual experiments) of unstimulated or LPS/U0126 stimulated mixed glial cell cultures were determined by quantitative real-time PCR. **(b)** IFNβ levels in the supernatant of unstimulated, LPS stimulated or LPS/U0126 stimulated mixed glial cells were determined by ELISA. **(c)** Ecto-ART activity on microglia from mixed glial cells was measured by using eNAD^+^/1G4 in response to 24 h stimulation with rising concentrations of IFNβ (1–1000 U). **(d)** Induction of ecto-ART activity upon IFNβ stimulation was compared in BALB/c WT and ARTC2.1^−/−^ microglia. **(e)** Upregulation of ARTC2.1 expression on microglia upon IFNβ stimulation was measured using an ARTC2.1-specific mAb in comparison to isotype control. Data are representative of 2–3 independent experiments. Statistical comparison of two groups was performed by using the student’s t test (p < 0.05 = */p < 0.01 = **/p < 0.001 = ***).

IFNβ has been described as a key cytokine driving the expression of ARTC2.1 in macrophages upon LPS/U0126 stimulation^8^. To test whether IFNβ is also expressed by LPS/U0126 stimulated microglia obtained from mixed glial cell cultures we first measured *Ifnb1* mRNA expression in sorted microglia from LPS/U0126 stimulated cultures. Here, we detected a significant upregulation of *Ifnb1* when compared to unstimulated controls (Fig. 3a). Further, we detected significantly increased levels of soluble IFNβ in the supernatants of these LPS/U0126 stimulated mixed glial cells (Fig. 3b). Next, we tested if IFNβ alone was able to induce ecto-ART activity in microglia. Indeed, IFNβ stimulated microglia exhibited a dose-dependent increase of cell surface eADP-ribosylation after incubation with eNAD^+^/DTT (Fig. 3c). The IFNβ induced ecto-ART activity was ARTC2.1-dependent since ARTC2.1^−/−^ microglia did not show any increase in ecto-ART activity after INFβ stimulation (Fig. 3d). Using specific monoclonal antibodies, we could confirm an increase in ARTC2.1 expression on IFNβ stimulated microglia, when compared to unstimulated controls (Fig. 3e). In summary, IFNβ induced ecto-ART activity on microglia by increasing the cell surface expression of ARTC2.1.

### Identification of ARTC2.1 target proteins on microglia by mass spectrometry

**Figure 4:**
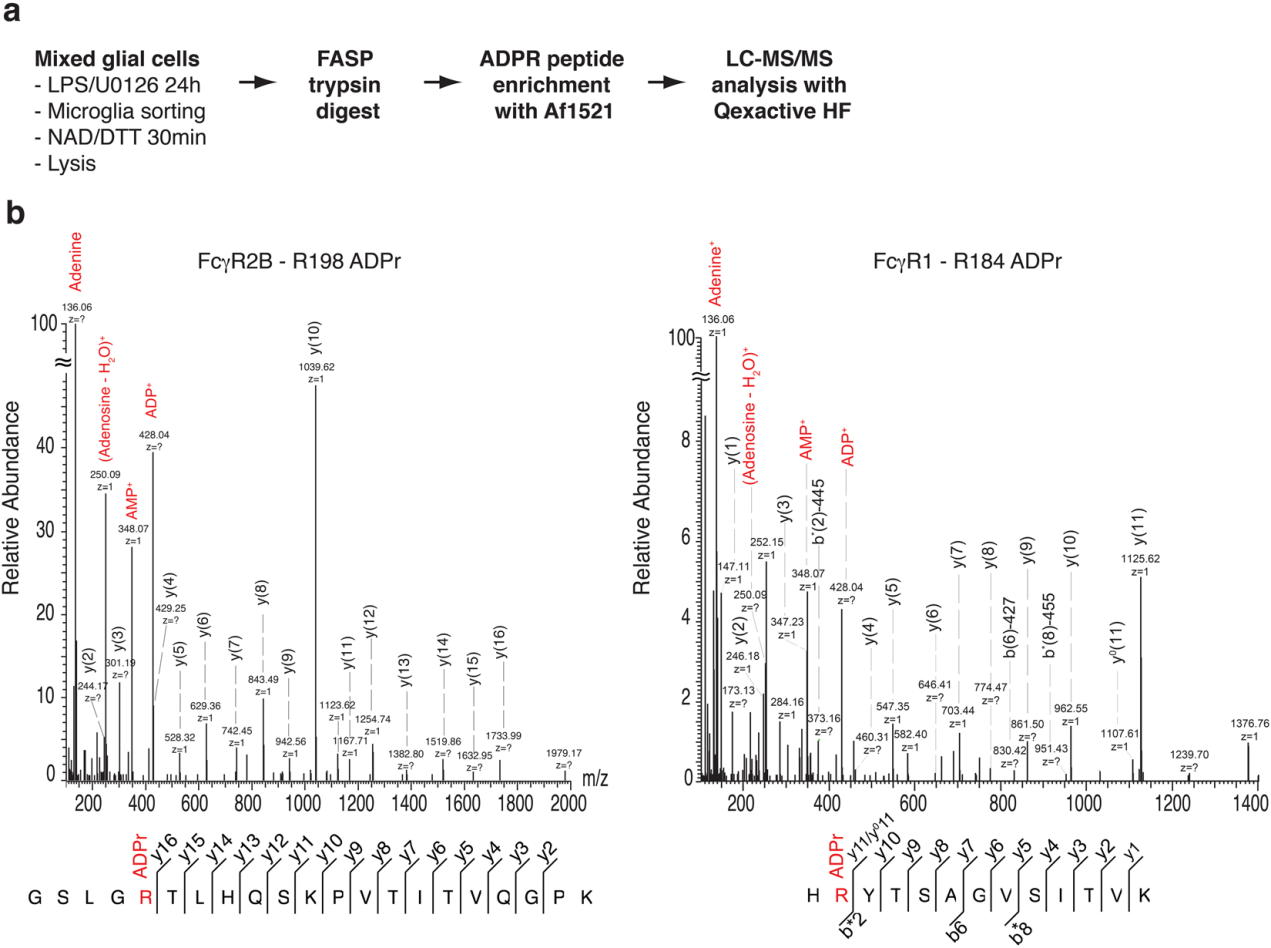
MS/MS analyses of ADP-ribosylated peptides fragmented using HCD. (**a)** Experimental setup for the identification of ARTC2.1 target proteins in microglia. **(b)** The presented peptides belong to FcγR2B, which is ADP-ribosylated at R198 (left) and FcγR1, which is ADP-ribosylated at R184 (right). The ADP-ribose specific fragment is indicated in the spectrum in red and peptide sequence coverage is shown below the respective spectrum.

In order to identify the targets of ARTC2.1 on microglia, we used a newly developed mass spectrometry-based strategy^18^. For this, microglia from LPS/U0126 treated WT and ARTC2.1^−/−^ mixed glial cell cultures were sorted by FACS and incubated with NAD^+^/DTT. Cells were lysed, peptides were generated from the isolated proteins by trypsin digestion and the ADP-ribosylated peptides enriched using the ADPR-binding macrodomain Af1521 (Fig. 4a). Subsequent mass spectrometry analyses (LC-MS/MS) revealed 54 unique, ADP-ribosylated peptides corresponding to 40 distinct cell surface target proteins on WT microglia (Table 1, Fig. 4b). Importantly, no ADP-ribosylated peptides were detected in samples of microglia from ARTC2.1^−/−^ mice, confirming the specificity of the method. Most identified targets were ADP-ribosylated on one site (30 targets), seven on two sites (Alcam, Clec12a, Enpp1, Icam1, Ptprc, Slc3a2, Tfrc), two on three sites (Fcgr2b, Itgb5) and one on four sites (Itgam). The majority of the detected ADP-ribosylated peptides were ADP-ribosylated on arginine residues (43 peptides), followed by glutamate (6 peptides) and aspartate (5 peptides) residues. Most of the newly identified ARTC2.1 targets are involved in cell-cell or cell-matrix interaction processes. However, some targets are also directly related to regulation of immune cell function such as detection of pathogen associated molecular pattern (CD14, Tlr2), phagocytosis (Mrc1), antigen presentation (H2-D1, H2-K1) or binding of immunoglobulines (FcεR1, FcγR1, FcγR2B).

**Table 1:** List of ADP-ribosylated peptides identified from LPS/U0126 stimulated microglia.

gene	protein	protein accession	ADPR site	ADPR AA	confidence	peptide sequence	peptide score	expect value
Alcam	CD166 antigen	tr|E9Q3Q6|E9Q3Q6_MOUSE	319	R	100%	(R)NMAASTTITVHYLDLSLNPSGEVTK	54	0,0015
Alcam	CD166 antigen	tr|E9Q3Q6|E9Q3Q6_MOUSE	386	D	99,85%	LRSSPSFSSLHYQ(D)AGNYVCETALQEVEGLK	74,34	2,20E-07
Bcam	Basal cell adhesion molecule	sp|Q9R069|BCAM_MOUSE	484	D	96,83%	LTWSQRG(D)TPAEPPFEGR	30,05	0,0031
Cd14	Monocyte differentiation antigen CD14	sp|P10810|CD14_MOUSE	68	R	100%	(R)VDTEADLGQFTDIIK	132,14	2,00E-11
Cd40	Isoform V of Tumor necrosis factor receptor superfamily member 5	sp|P27512-5|TNR5_MOUSE	247	E	99,83%	ISVQ(E)RQVTDSIALRPLV	52,58	1,20E-05
Cd9	CD9 antigen	sp|P40240|CD9_MOUSE	131	R	96,99%	L(R)SKDEPQR	27,61	0,0053
Clec12a	C-type lectin domain family 12 member A	sp|Q504P2|CL12A_MOUSE	197	D	100%	K(D)RTQYPLSEK	32,74	0,0018
Clec12a	C-type lectin domain family 12 member A	sp|Q504P2|CL12A_MOUSE	215	R	97,79%	MFLSEESE(R)STDDIDKK	27,02	0,0064
Enpp1	Ectonucleotide pyrophosphatase/phosphodiesterase 1 isoform CRA_d	tr|G3 × 9S2|G3 × 9S2_MOUSE	853	R	100%	(R)ESSWVEELLTLHR	96,1	7,40E-08
Enpp1	Ectonucleotide pyrophosphatase/phosphodiesterase 1 isoform CRA_d	tr|G3 × 9S2|G3 × 9S2_MOUSE	854	E	99,31%	R(E)SSWVEELLTLHR	63,12	1,50E-06
Fcer1g	High affinity immunoglobulin epsilon receptor subunit gamma	sp|P20491|FCERG_MOUSE	71	R	100,00%	EKADAVYTGLNT(R)SQETYETLK	76,36	7,90E-06
Fcgr1	High affinity immunoglobulin gamma Fc receptor I	sp|P26151|FCGR1_MOUSE	184	R	100%	H(R)YTSAGVSITVK	40,29	0,00025
Fcgr2b	Low affinity immunoglobulin gamma Fc region receptor II	tr|E9Q415|E9Q415_MOUSE	198	R	100,00%	GSLG(R)TLHQSKPVTITVQGPK	107,45	9,70E-11
Fcgr2b	Low affinity immunoglobulin gamma Fc region receptor II	tr|E9Q415|E9Q415_MOUSE	88	R	100%	SI(R)SQVQASYTFK	61,23	1,80E-06
Fcgr2b	Low affinity immunoglobulin gamma Fc region receptor II	tr|E9Q415|E9Q415_MOUSE	158	R	100%	LLN(R)ISFFHNEK	30,35	0,0016
H2-D1	Isoform 2 of H-2 class I histocompatibility antigen alpha chain	sp|P01895-2|HA1Y_MOUSE	7	R	100%	A(R)WIEQEGPEYWER	77,61	4,10E-06
H2-K1	H-2 class I histocompatibility antigen K-D alpha chain	sp|P01902|HA1D_MOUSE	169	E	98,50%	RKW(E)QAGDAEYYR	31,46	0,0011
Hfe	Hemochromatosis isoform CRA_a	tr|Q5SZ87|Q5SZ87_MOUSE	120	E	100%	LL(E)LGRGVLGQQVPTLVK	47,31	7,20E-05
Icam1	Isoform 2 of Intercellular adhesion molecule 1	sp|P13597-2|ICAM1_MOUSE	345	R	99,86%	VVLLSGVEP(R)PPTPQVQFTLNASSEDHKR	35,05	0,00054
Icam1	Isoform 2 of Intercellular adhesion molecule 1	sp|P13597-2|ICAM1_MOUSE	152	R	99,02%	GEEILS(R)QPVGGHPK	27,87	0,0057
Icoslg	Isoform 2 of ICOS ligand	sp|Q9JHJ8-2|ICOSL_MOUSE	108	R	100%	N(R)GHLSLDSMK	30,38	0,0029
Il2rg	Cytokine receptor common subunit gamma	sp|P34902|IL2RG_MOUSE	196	R	100%	D(R)SWTELIVNHEPR	41,46	0,00013
Itga5	Integrin alpha-5	sp|P11688|ITA5_MOUSE	903	R	100%	EAPG(R)SSTASGTQVLK	25,81	0,0064
Itga6	Integrin alpha-6 (Fragment)	tr|F6VSK8|F6VSK8_MOUSE	188	R	100%	LRPIPITASVEIQEPSS(R)	69,51	2,70E-05
Itgam	Integrin alpha-M	tr|Q3U1U4|Q3U1U4_MOUSE	415	R	100%	N(R)VQSLVLGAPR	51,11	2,00E-05
Itgam	Integrin alpha-M	tr|Q3U1U4|Q3U1U4_MOUSE	502	R	100%	A(R)WQCEALLHGDQGHPWGR	94,4	1,20E-07
Itgam	Integrin alpha-M	tr|Q3U1U4|Q3U1U4_MOUSE	277	R	99,18%	FGDPLDYKDVIPEAD(R)AGVIR	50,86	4,20E-05
Itgam	Integrin alpha-M	tr|Q3U1U4|Q3U1U4_MOUSE	506	E	95,00%	ARWQC(E)ALLHGDQGHPWGR	30,38	0,0017
Itgav	Integrin alpha-V light chain	tr|A2AKI5|A2AKI5_MOUSE	855	R	100%	DLTL(R)EGDVHTLGCGIAK	60,04	0,00031
Itgb1	Isoform 2 of Integrin beta-1	sp|P09055-2|ITB1_MOUSE	228	R	100%	NVLSLTD(R)GEFFNELVGQQR	55,9	7,50E-06
Itgb2	Integrin beta	tr|Q542I8|Q542I8_MOUSE	454	R	100%	DQS(R)EQSLCGGK	41,49	0,0002
Itgb5	Integrin beta	tr|Q6PE70|Q6PE70_MOUSE	226	R	100%	HLLPLTD(R)VDSFNEEVRK	30,98	0,0013
Itgb5	Integrin beta	tr|Q6PE70|Q6PE70_MOUSE	72	R	100%	ANLI(R)NGCEGEIESPASSTHVLR	84,68	1,30E-06
Itgb5	Integrin beta	tr|Q6PE70|Q6PE70_MOUSE	158	R	99,85%	DDLENI(R)SLGTK	39,92	0,031
Lgals1	Galectin-1	sp|P16045|LEG1_MOUSE	116	E	99,97%	FPNRLNM(E)AINYMAADGDFK	42,7	0,00015
Lrpap1	Alpha-2-macroglobulin receptor-associated protein	sp|P55302|AMRP_MOUSE	71	R	100%	(R)LHLSPVR	20,46	0,012
Mcam	Isoform 2 of Cell surface glycoprotein MUC18	sp|Q8R2Y2-2|MUC18_MOUSE	71	R	100%	E(R)QILIFR	39,14	0,0066
Mrc1	Macrophage mannose receptor 1	sp|Q61830|MRC1_MOUSE	545	R	100,00%	AYLTTVED(R)YEQAFLTSLVGLRPEK	85,03	1,10E-08
Pdia3	Protein disulfide-isomerase A3	sp|P27773|PDIA3_MOUSE	62	R	100%	(R)LAPEYEAAATR	63,08	0,00013
Ptprc	Receptor-type tyrosine-protein phosphatase C	tr|S4R1M0|S4R1M0_MOUSE	168	R	100%	(R)NTFIPER	24,41	0,0051
Ptprc	Receptor-type tyrosine-protein phosphatase C	tr|S4R1M0|S4R1M0_MOUSE	508	R	100%	N(R)YVDILPYDYNR	42	0,015
Ptprj	Receptor-type tyrosine-protein phosphatase eta	sp|Q64455|PTPRJ_MOUSE	202	R	100%	(R)IPVTNLSQLHK	29,43	0,0017
Pvrl2	Isoform Alpha of Nectin-2	sp|P32507-2|PVRL2_MOUSE	104	R	100%	A(R)PETNADLR	20	0,037
Sdc3	Syndecan-3	sp|Q64519|SDC3_MOUSE	362	D	98,15%	GARPGPGLH(D)NAIDSGSSAAQLPQK	45,2	0,00037
Sdc4	Syndecan-4	sp|O35988|SDC4_MOUSE	116	R	100%	(R)APSDVGDDMSNK	35,64	0,023
Slamf7	Isoform 4 of SLAM family member 7	sp|Q8BHK6-4|SLAF7_MOUSE	77	R	100%	E(R)IVFPDGLYSMK	20,04	0,013
Slc3a2	Isoform 2 of 4F2 cell-surface antigen heavy chain	sp|P10852-2|4F2_MOUSE	509	R	100%	SA(R)LGASNLPAGISLPASAK	52,26	0,00024
Slc3a2	Isoform 2 of 4F2 cell-surface antigen heavy chain	sp|P10852-2|4F2_MOUSE	168	R	99,99%	IGDLQAFVG(R)DAGGIAGLK	46,05	0,0073
Slc9a1	Sodium/hydrogen exchanger 1	sp|Q61165|SL9A1_MOUSE	622	R	100%	AVTSD(R)ILPALSK	33,64	0,005
Stx4	Syntaxin-4	sp|P70452|STX4_MOUSE	192	D	99,99%	(D)TQVTRQALNEISAR	52,29	2,90E-05
Tfrc	Transferrin receptor protein 1	sp|Q62351|TFR1_MOUSE	658	R	100%	ATS(R)LTTDFHNAEK	31,69	0,007
Tfrc	Transferrin receptor protein 1	sp|Q62351|TFR1_MOUSE	358	R	100%	MEGSCPA(R)WNIDSSCK	41,06	0,029
Tlr2	Toll-like receptor	tr|G3 × 8Y8|G3 × 8Y8_MOUSE	187	R	100%	ALSL(R)NYQSQSLK	32,78	0,0011
Vamp3	Vesicle-associated membrane protein 3	sp|P63024|VAMP3_MOUSE	18	R	100%	(R)LQQTQNQVDEVVDIMR	57,88	0,00054

Mixed glial cells were stimulated with LPS (0.1 µg/ml) and U0126 (10 µM) overnight in BME medium containing 10% FCS. CD11b^+^ microglia were FACS sorted, incubated with 50 µM NAD and 2.5 mM DTT for 30 min on ice, washed twice with PBS and lysed using RIPA buffer. After trypsin digestion ADPR peptides were enriched using macrodomain Af1521 and samples were subjected to mass spectrometry analyses. Only peptides of cell surface proteins displaying a peptide score >20 and a confidence of >95% were included into the analyses.

### ADP-ribosylation of FcγR1 and FcγR2B diminishes binding of IgG

**Figure 5:**
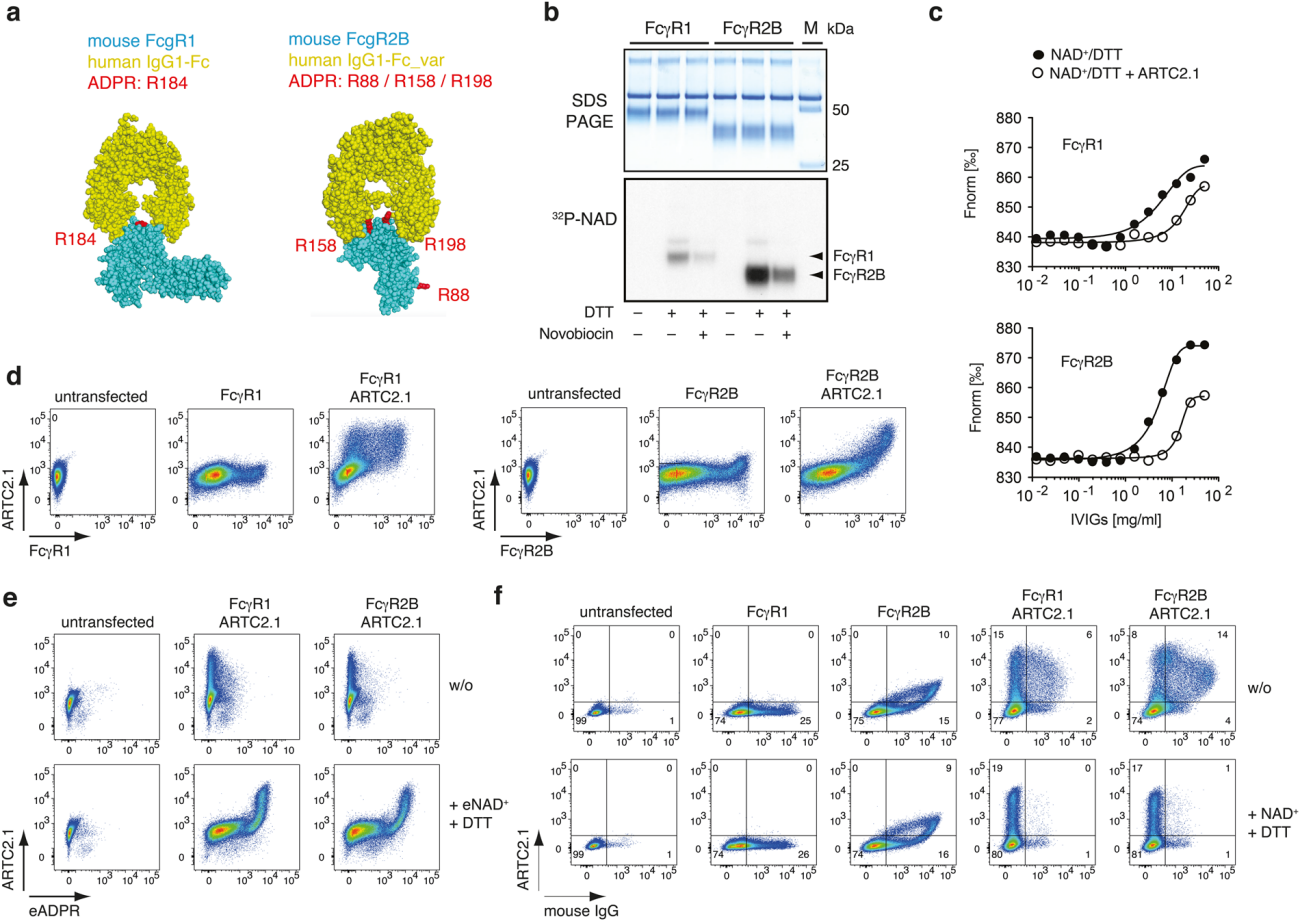
ADP-ribosylation of FcγRs affects binding of IgG to transfected HEK cells. (**a**) Using SWISS-MODEL, the structures of mouse FcγR1 and FcγR2B 3D structures were modeled on the corresponding structures of human FcγR1 (pdb: 4w4o) and FcγR2B (pdb: 3wjj) in complex with human IgG1-Fc. FcγRs are displayed in cyan, the Fc portion of IgG1 is shown in yellow. ARTC2.1 target arginines are labeled in red. (**b**) Soluble FcγR1 and FcγR2B were radiolabeled with ^32^P-NAD^+^ (1 µM) by soluble ARTC2.1 in the presence or absence of DTT (2.5 mM) and the ARTC inhibitor novobiocin. Proteins were fractioned by SDS-PAGE and subjected to autoradiography (cropped). (**c**) Binding of human intravenous immunoglobulins (IVIGs) to ADP-ribosylated or unmodified FcγR1 and FcγR2B was measured in a thermophoresis assay starting with an IVIG concentration of 50 mg/ml and 1:2 dilution steps. Each dot represents the mean of 4 measurements. (**d**) HEK cells were transfected with FcγR1 or FcγR2B alone or in combination with ARTC2.1. **(e)** Ecto-ART activity in FcγRs/ARTC2.1 co-transfected HEK cells was measured after incubation with or without eNAD^+^/DTT. **(f)** HEK cells transfected with FcγR1/FcγR2B or co-transfected with ARTC2.1 were pre-incubated with or without 100 µM NAD^+^ and 2 mM DTT. Cells were washed and incubated with BALB/c mouse serum (1:20 diluted) and cell surface binding of mouse IgG was measured using a PE-conjugated anti-mouse IgG F(ab)_2_. Data are representative of 2–3 independent experiments.

We were particularly intrigued by the discovery of two immunoglobulin G (IgG) receptors FcγR1 and FcγR2B as targets of ARTC2.1 and set out to address the question whether ARTC2.1-mediated modification of these proteins impinges on their function. *In silico* structural analyses of the observed ADP-ribosylation sites of FcγR1 (R184) and FcγR2B (R158, R198) revealed that these sites lie in close proximity to the binding site of the Fc portion of IgG, suggesting a potential influence of ADP-ribosylation at these sites on IgG binding (Fig. 5a **)**. To test this possibility, we established a microscale thermophoresis assay for IgG binding to soluble FcγR1 and FcγR2B. First, we validated the ADP-ribosylation of both soluble FcγRs by SDS-PAGE autoradiography after co-incubation with soluble ARTC2.1 and ^32^P-NAD^+^/DTT. The results confirmed that ARTC2.1 ADP-ribosylates both FcγRs and that this was inhibited by the ARTC inhibitor novobiocin (Fig. 5b). Interestingly, FcγR2B showed a more pronounced ADPR signal compared to FcγR1, consistent with ADP-ribosylation on more than one site. We then used highly concentrated human intravenous immunoglobulins (IVIGs), known to bind to murine FcγR1 and FcγR2B^23^, and probed the impact of ADP-ribosylation on IgG binding to FcγRs. When FcγRs were preincubated with NAD^+^/DTT in the presence of ARTC2.1, microscale thermophoresis analysis indeed revealed a 3.2-fold diminished IgG binding to FcγR1 and a 2.5-fold reduced binding to FcγR2B (Fig. 5c). To strengthen this finding, we transiently transfected human embryonic kidney (HEK) cells with FcγR1 or FcγR2B expression plasmids alone or in combination with an ARTC2.1 expression plasmid (Fig. 5d). FcγR/ARTC2.1 co-transfected HEK cells exhibited robust cell surface ADP-ribosylation upon incubation with eNAD^+^/DTT (Fig. 5e). Of note, HEK cells were incubated with or without NAD^+^/DTT to induce cell surface ADP-ribosylation prior to addition of diluted IgG-containing mouse serum. Moreover, we did not detect any impact of the reducing agent DTT on IgG binding to FcγR1 and FcγR2B single transfected HEK cells (Fig. 5f). Co-transfection of FcγR1 or FcγR2B with ARTC2.1, however, led to an almost complete reduction of IgG binding to the HEK cells when cells were pretreated with NAD^+^/DTT, confirming that ADP-ribosylation of FcγR1 and FcγR2B diminishes IgG binding. In summary, we confirmed FcγR1 and FcγR2B as targets of ARTC2.1-mediated ADP-ribosylation and demonstrated that the covalent attachment of the ADP-ribose group to both FcγRs impinges on their binding to IgG.

### Cell surface ADP-ribosylation on microglia diminishes IgG binding and phagocytosis of IgG-coated beads

**Figure 6:**
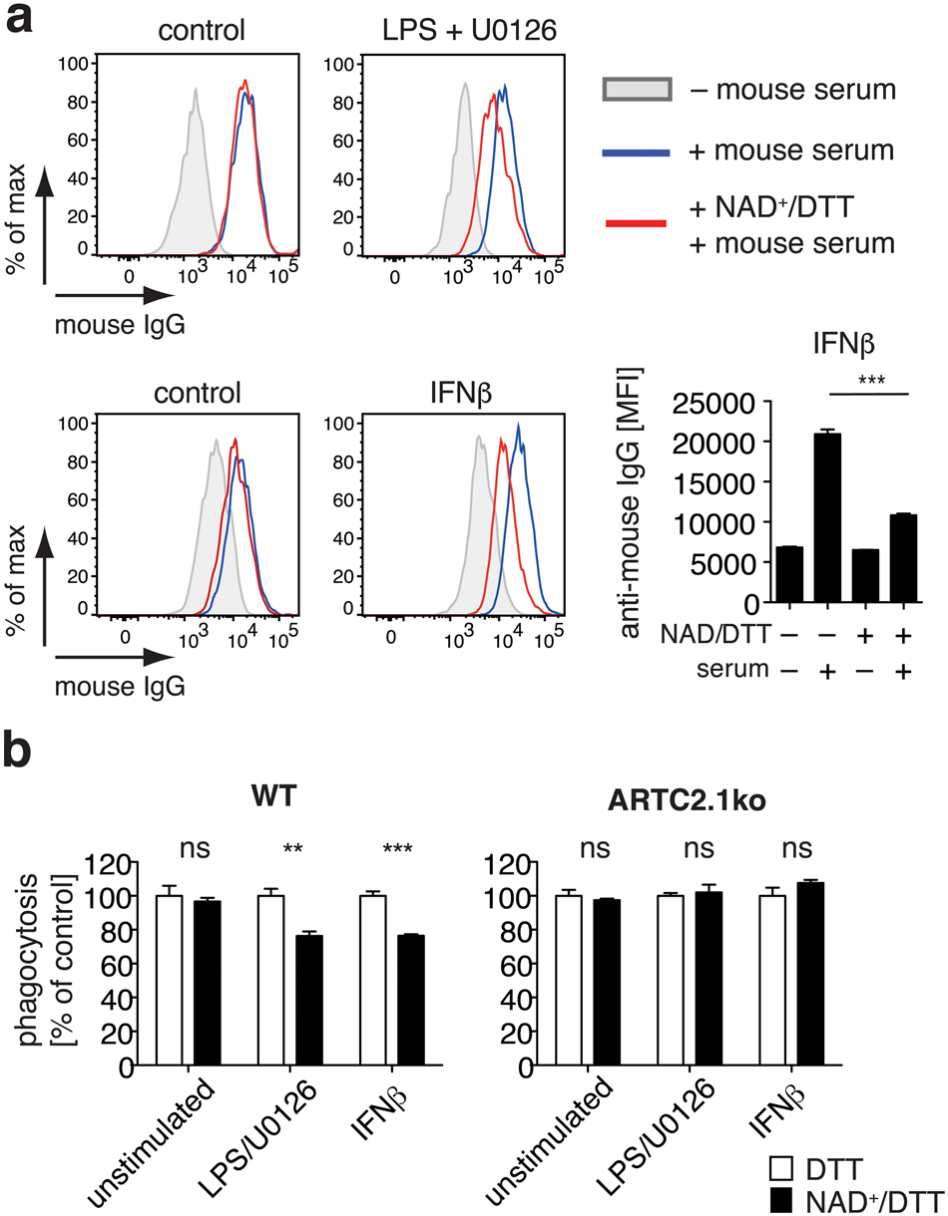
ADP-ribosylation of FcγRs affects binding of IgG to microglia. (**a**) IgG binding to unstimulated, LPS/U0126-stimulated (upper plot) or IFNβ-stimulated (lower plot) microglia was analyzed by flow cytometry. Cells were pre-incubated with NAD^+^/DTT or left untreated, washed twice with PBS and were incubated with IgG containing mouse serum (1:20 diluted) for 30 min. Cells were washed twice and cell surface binding of mouse IgG was measured using a PE-conjugated anti-mouse IgG F(ab)_2_. **(b)** Phagocytosis of PE-labeled IgG-coated latex beads by unstimulated, LPS-stimulated or IFNβ-stimulated microglia from WT or ARTC2.1^−/−^ mice was measured by flow cytometry. Cells were either preincubated with DTT alone or with NAD^+^/DTT for 30min, washed twice and allowed to phagocytose IgG-beads for 3h. Phagocytosis rate was normalized to the corresponding DTT control sample (100%).  Data are representative of 2–3 independent experiments. Statistical comparison of two groups was performed by using the student’s t test (p < 0.05 = */p < 0.01 = **/p < 0.001 = ***).

We next analyzed the influence of cell surface ADP-ribosylation on IgG binding by LPS/U0126 or IFNβ treated microglia. For this, we incubated LPS/U0126 stimulated, IFNβ stimulated or unstimulated mixed glial cells with IgG-containing mouse serum and analyzed binding of IgG to these cells by flow cytometry using fluorochrome-conjugated F(ab)_2_ anti-mouse IgG. We observed a reduced binding of murine IgG to either LPS/U0126 or IFNβ treated microglia when cells were pre-incubated with NAD^+^/DTT (Fig. 6a). Conversely, unstimulated microglia did not exhibit a comparable shift in IgG binding upon pre-incubation with NAD^+^/DTT.

Finally, we probed the impact of microglia cell surface ADP-ribosylation on FcγR-mediated phagocytosis. For this, we stimulated WT microglia with LPS/U0126 or IFNβ for 24 h to induce ecto-ART activity. Cells were then incubated for 30 min with NAD^+^/DTT or with DTT alone to exclude a potential impact of the reducing agent DTT on the phagocytic capacity of the microglia. We then washed the cells and added PE-labeled IgG-coated latex beads to the culture. The analyses of phagocytosis by flow cytometry revealed that the NAD^+^/DTT incubation diminished phagocytosis of IgG beads by LPS/U0126 or IFNβ stimulated microglia by about 25% when compared to unstimulated controls (Fig. 6b). This effect was virtually absent in ARTC2.1^−/−^ microglia treated in a similar fashion. Together, these findings suggest a functional role of ARCT2.1-mediated ADP-ribosylation of FcγRs on microglia by regulating IgG-dependent phagocytosis.

## Discussion

In this study we demonstrated that murine microglia express ecto-ADP-ribosyltransferase ARTC2.1, which is strongly upregulated under LPS treatment and simultaneous inhibition of the ERK1/2 signaling pathway or by stimulation with IFNβ. In the presence of NAD^+^ and reducing conditions this leads to the ARTC2.1-dependent ADP-ribosylation of numerous cell surface proteins. We used mass spectrometry to search for new ARTC2.1 targets and identified 40 ADP-ribosylated cell surface proteins on microglia, most of them being associated with microglia immune function such as adhesion, sensing of pathogen associated molecular patterns, antigen presentation and immunoglobulin binding. In a proof-of-principle experiment we were able to show that ADP-ribosylation of FcγR1 and FcγR2B functionally inhibited cell surface IgG binding and phagocytosis of IgG-coated beads in microglia.

ARTC2 expression in murine immune cells has been reported in previous studies. Antigen presenting cells such as macrophages and dendritic cells preferentially express ARTC2.1, whereas T cells can express both, ARTC2.1 and ARTC2.2^22^. Studies have shown that bone marrow-derived macrophages (BMDM) strongly upregulate cell surface ARTC2.1 upon proinflammatory stimulation with LPS in combination with ERK1/2 inhibition^8^. One proposed mechanism underlying ARTC2.1 upregulation in BMDM is the release of IFN-β, which stimulates BMDM in an autocrine fashion. Indeed, we could demonstrate that LPS/U0126 stimulation induces IFNβ expression in microglia and that microglia stimulated with IFNβ alone exhibit a pronounced, ARTC2.1-dependent, ecto-ART activity. Another recent study has shown that injection of the Toll-like receptor 3 agonist polyI:C induced IFNβ expression in microglia *in vivo*. This suggests that proinflammatory triggers could potentially induce upregulation of ARTC2.1 in microglia *in vivo* ^24^.

The upregulation of ARTC2.1 on microglia under inflammatory conditions could represent a new way of fine-tuning microglia immune responses. However, ARTC2.1 needs reducing conditions to unlock its enzymatic activity. Extracellular antioxidants such as glutathione or ascorbic acid have been found in rodent brains^25^ and therefore potentially could serve as reducing agents for ARTC2.1 expressed on brain microglia. Interestingly, release of antioxidants is upregulated within 1–2 h after brain ischemia^25^, a condition resulting in massive tissue necrosis that leads to sterile inflammation^26^. Both, cell damage and inflammation, also enable the release of NAD^+^ into the extracellular space where it could serve as substrate for ARTC2.1^27,28^. Therefore, future experiments should investigate the expression of ARTC2.1 on microglia during brain ischemia. Based on the present findings, ARTC2.1 could potentially modulate the microglia response to sterile inflammation by ADP-ribosylating microglia cell surface proteins involved in immune functions.

To identify potential targets of ARTC2.1 on microglia, we used a novel mass spectrometry based approach that includes enrichment of ADP-ribosylated peptides via the ADP-ribose binding macrodomain Af1521. With this approach we identified 40 cell surface proteins that were ADP-ribosylated in the presence of NAD^+^/DTT. Their ADP-ribosylation was strictly dependent on the enzymatic activity of ARTC2.1 as we could not detect any ADP-ribosylated peptides derived from ARTC2^−*/*−^ microglia. However, it is worth noting that even though our novel approach provided important information regarding ARTC2.1 protein targets in microglia, we cannot exclude that some ADP-ribosylated proteins were not detected using this technique. For instance, the P2X7 receptor (*P2rx7*), a known target for ARTC2-mediated ADP-ribosylation on macrophages and on T cells^29^, is expressed on microglia (Fig. S1a), but the corresponding ADP-ribosylated peptide was not detected using our mass spectrometry method. In contrast, immunoprecipitation of P2X7 from ^32^P-NAD^+^/DTT treated microglia and subsequent autoradiography confirmed that P2X7 is also a target for ADP-ribosylation on microglia (Fig. S1b). Hence, even though enrichment of ADP-ribosylated peptides by macrodomain Af1521 and subsequent mass spectrometry analyses displayed a very powerful approach to define a cell surface “ADP-ribosylome”, some ADP-ribosylated target proteins could be left unnoticed due to technical constraints.

Further investigations are needed to define the functional impact of ADP-ribosylation on the newly identified ARTC2.1 targets on microglia. It is likely that ADP-ribosylation impairs the function of some of the newly identified targets. Recently we could show that ADP-ribosylation of the interleukin 2 (IL-2) receptor alpha chain (CD25) on regulatory T cells (Tregs) affects the binding of IL-2 and thereby diminished Treg STAT5 phosphorylation and proliferation^15^. In a proof-of-principle experiment we evaluated the impact of ADP-ribosylation on immunoglobulin binding by FcγRs on microglia, since the ADP-ribosylation sites we identified were in close proximity to the receptor binding sites for IgG Fc fragments. Indeed, we observed that ADP-ribosylation of FcγR1 and FcγR2B affected IgG binding to microglia. FcγR1 and FcγR2B have opposing biological roles in microglia: the high affinity FcγR1 triggers microglia activation and promotes phagocytosis and the release of inflammatory mediators. Conversely, the low affinity FcγR2B elicits counteracting inhibitory signals^30^. Since ADP-ribosylation reduced IgG binding to both FcγRs, the overall biological effect of ADP-ribosylation on microglial FcγRs under inflammatory conditions in the brain (e.g. ischemic stroke) needs to be further investigated. Interestingly, FcγR^−/−^ mice show significantly reduced microglia activation and smaller infarct sizes compared to WT mice in the middle cerebral artery occlusion (MCAO) mouse model for ischemic stroke^31^. Therefore, it is conceivable that ADP-ribosylation of FcγR could represent a protective physiological mechanism to control the activation of microglia during sterile inflammation in mice. However, it must be noted that the direct transfer of this newly discovered mechanism from mouse to human microglia remains unclear, as functional ARTC2 does not exist in humans due to a premature stop codon^32^. Therefore, it will be necessary to analyze human microglia for the expression of other cell surface ADP-ribosyltransferases that could serve to mediate cell surface ART activity.

In conclusion, we identified ARTC2.1 as major ecto-ART expressed by microglia and identified multiple new ADP-ribosylation targets of the microglia cell surface by mass spectrometry thus defining a microglia cell surface “ADP-ribosylome”. It will be necessary to further analyze the functional impact of ADP-ribosylation on each identified target, as we did in a proof-of-principle experiment for FcγRs. This will provide further insights into the regulatory role of this post-translational modification during inflammation. Finally, our approach could be used to define the cell surface “ADP-ribosylome” of other ARTC2-expressing immune cell populations such as T cells.

## Material and Methods

### Mice

ARTC2^−/−^, ARTC2.1^−/−^ and ART2.2^−/−^ mice^33^ were backcrossed onto the BALB/c background for at least 12 generations. All mice were bred at the animal facility of the University Medical Center (UKE). All experiments involving tissue derived from animals were performed with approval of the responsible regulatory committee (Hamburger Behörde für Gesundheit und Verbraucherschutz, Veterinärwesen/Lebensmittelsicherheit, ORG-722). All methods were performed in accordance with the relevant guidelines and regulations.

### Mixed glial cell cultures and stimulation with LPS/U0126 or IFNβ

The brains of 1 to 2 days old neonatal mice were prepared and transferred into Hanks Balanced Salt Solution (HBSS, ThermoFisher) containing 10 mM HEPES (ThermoFisher). After removal of the meninges, brains were minced, washed and incubated for 25 min in HBSS + 10 mM HEPES with 0.5 mg/ml papain (Sigma-Aldrich) and 10 μg/ml DNAse (Roche Diagnostics). Cells were washed again in BME medium (Life Technologies), dissociated and then plated at a density of 3 × 10^5^ cells/ml and cultured in BME media supplemented with 10% FCS and penicillin/streptomycin. Cells were used for analyses after 21 days of culture and contained 20–30% microglia and 60–70% astrocytes. Cells were stimulated with LPS (0.1 µg/ml, Sigma-Aldrich) and U0126 (10 µM, Sigma-Aldrich) or with 1–1000 U/ml recombinant mouse IFNβ (BioLegend) for 24 h before ADP-ribosylation assays.

### Antibodies and flow cytometry

Cells were analyzed using BD FACSCanto II following staining with fluorochrome-conjugated mAbs: anti-CD3 (clone 145-2C11, Biolegend), anti-ARTC2.1 (clone R18A136#2; UKE), anti-ARTC2.2 (clone Nika109; UKE), anti-etheno-ADP-ribose (clone 1G4, UKE)^34^, anti-CD11b (clone M1/70; BioLegend), anti-GLAST (clone ACSA-1; Miltenyi), anti-FcγR1 (clone  X54-5/7.1, BioLegend) and anti-FcγR2B (clone AT130-2, eBioscience), anti-P2X7 (clone RH23A44, UKE), donkey anti-mouse IgG F(ab)_2_ (Dianova). For flow cytometric analyses, spleen T cells were identified as CD3^+^, spleen macrophages as F4/80^+^ cells. Microglia were identified as CD11b^+^GLAST^−^ cells and astrocytes as CD11b^−^GLAST^+^. For the detection of mouse IgG on the cell surface of the microglia or transfected HEK cells, cells were incubated with fresh BALB/c mouse serum (1:20 dilution), washed twice and IgG binding was detected with a PE-conjugated donkey-anti mouse IgG F(ab)_2_.

For some experiments microglia and astrocytes were sorted at the FACS Core Facility at the University Medical Center Hamburg-Eppendorf (UKE) on a BD FACSAria III. Analysis of flow cytometric data was performed using FlowJo (Miltenyi Biotec).

### Etheno-ADP-ribosylation assay

Cultured glial cells or splenocytes were incubated for 20 min at 4 °C with 100 µM etheno-nicotinamide adenine dinucleotide (eNAD^+^, Sigma-Aldrich) in the presence or absence of 2–5 mM dithiothreitol (DTT, Invitrogen). eNAD^+^ was then removed by washing cells twice with PBS, 1% fetal calf serum (FCS, Gibco). Etheno-ADP-ribose incorporated into cell surface proteins was detected as described previously^20^ using fluorochrome-conjugated etheno-adenosine-specific monoclonal antibody 1G4. Cells were washed with PBS, 1% FCS and analyzed by flow cytometry. Cells that were not treated with eNAD^+^ were stained with 1G4 and used as control.

### ^32^P-NAD^+^ ADP-ribosylation assay

Radio-ADP-ribosylation of cell surface proteins was performed as described previously^15,27^ using either mixed cultured glial cells or FACS-sorted microglia or astrocytes. Cells were incubated with 1 µM ^32^P-NAD^+^ for 15 min at 37 °C in the absence or presence of 5 mM DTT. Cells were washed 4 times at 4 °C in PBS and cell membrane proteins were then solubilized with 1% Triton X-100 in PBS containing 2 µM PARP-inhibitor PJ-34 (ENZO Life Sciences) for 30 min at 4 °C. Cell lysates were clarified from nuclei and other insoluble proteins by high-speed centrifugation. Proteins were size fractionated by SDS-PAGE and stained with Coomassie, radiolabeled proteins were detected by autoradiography.

### Quantitative real-time PCR

RNA was extracted from FACS sorted microglia using RNeasy® Plus Mini Kit (Qiagen) followed by cDNA synthesis using the Maxima First Strand cDNA Synthesis Kit (Thermo Fisher Scientific) as recommended by the respective supplier.

Taqman probes (ThermoFisher Scientific) for mouse *Art2a* (Mm01269252_m1) and mouse *Ifnb1* (Mm00439552_s1) were used for quantitative real-time PCR and measured on a Lightcycler 96 (Roche). The relative gene expression was calculated using the ΔΔCt method. Samples were normalized to the expression of the housekeeping gene *Sdha* (Mm01352366_m1). Untreated samples were used as calibrator and compared to LPS/U0126 stimulated samples.

### IFNβ ELISA

IFNβ secretion by mixed glial cells upon stimulation with LPS/U0126 was measured using a commercially available mouse IFNβ ELISA kit (Biolegend). ELISA was performed according to the manufacturer’s instructions.

### Enrichment of ADP-ribosylated peptides

Microglia cells were lysed in RIPA buffer (Sigma-Aldrich), supplemented with Roche complete (1x), Pefa (AEBSF, 4 µM, Sigma-Aldrich), Novobiocin (200 µM, Sigma-Aldrich) and PJ34 (PARP inhibitor, 2 µM, Sigma-Aldrich). To process and digest the samples we used trypsin digestion and a filter-aided sample preparation (FASP) method. Samples containing 250 µg proteins were diluted and reduced in Urea Buffer (8 M Urea, 0.1 M Tris-HCl pH 8) containing 1 mM DTT, transferred to a Microcon-30-kDa-cutoff centrifugal filter unit (Millipore), centrifuged at 14,000 g at 20 °C for 20 min and washed with 200 µl urea buffer. Samples were alcylated using urea buffer containing 20 mM chloroacetamide and washed two times with 100 μl urea buffer and three times with 100 µl 50 mM ammonium bicarbonate. The on filter digestion was performed in 50 mM ammonium bicarbonate using a 1:50 protein to sequencing grade modified trypsin (Promega) ratio overnight at room temperature.

ADP-ribosylated peptide enrichment was performed as previously described^18^. The peptide mixture was diluted in IP buffer (50 mM Tris–HCl, pH 8, 10 mM MgCl_2_, 250 μM DTT and 50 mM NaCl) and binding was carried out for 2 h at 4 °C using the Af1521 macrodomain GST-fusion protein coupled to glutathione-Sepharose beads. Beads were washed three times with IP buffer and bound peptides were eluted with 0.15% TFA. The resulting mixture was desalted using stage tips packed with C18 filters.

### Liquid chromatography and mass spectrometry analysis

All data were acquired on an Orbitrap Q-Excative HF mass spectrometer (Thermo Fisher Scientific) connected to a nano EasyLC 1000 HPLC system (Thermo Fisher Scientific). 4 µl of peptide sample in 0.1% formic acid and 3% acetonitrile are loaded onto a self-made column (75 µm × 150 mm) packed with reverse-phase C18 material (ReproSil-Pur 120 C18-AQ, 1.9 µm, Dr. Maisch GmbH) and separated at a flow rate of 300 nL/min by a gradient from 2 to 25% B in 90 minutes. Solvent composition at the channels A and B was 0.1% formic acid and 0.1% formic acid, 99.9% acetonitrile, respectively. The mass spectrometer was set to acquire full-scan MS spectra (300–1700 m/z) at a resolution of 60’000 after accumulation to an automated gain control (AGC) target value of 3e6. Charge state screening was enabled, and unassigned charge states, and single charged precursors were excluded. Ions were isolated using a quadrupole mass filter with a 2 m/z isolation window. A maximum injection time of 240 ms was set. HCD fragmentation was performed at a normalized collision energy (NCE) of 28%. Selected ions were dynamically excluded for 20 sec.

### Database Analyses and Configuration of Mascot Modifications

The raw file processing and the database search using Mascot was performed as described in^35^. Briefly, MS and MS/MS spectra were converted to Mascot generic format (MGF) using Proteome Discoverer, v2.1 (Thermo Fisher Scientific). The MGFs were searched against the UniProtKB mouse database (taxonomy 10090, version 20160902), which included 24905 Swiss-Prot, 34616 TrEMBL entries, 59783 decoy hits, and 262 common contaminants. Mascot 2.5.1.3 (Matrix Science) was used for peptide sequence identification. A peptide tolerance was set to 10 ppm and MS/MS tolerance was 0.05 Da. Enzyme specificity is set to trypsin, allowing up to 4 missed cleavages. The ADP-ribose variable modification was set to a mass shift of 541.0611, with scoring of the neutral losses equal to 347.0631 and 249.0862. The marker ions at m/z 428.0372, 348.0709, 250.0940, 136.0623 are ignored for scoring. R, K, D and E are set as variable ADP-ribose acceptor sites. Carbamidomethylation is set as a fixed modification on C and oxidation as a variable modification on M. Peptides are considered correctly identified when a Mascot score >20 and an expectation value <0.05 are obtained.

### Microscale thermophoresis assay for IgG binding to FcγRs

To evaluate the impact of ADP-ribosylation on FcγRs, we used microscale thermophoresis to quantify biomolecular interactions. We labeled recombinant FcγR1 (Sino Biological) and FcγR2B (Sino Biological) with Monolith Protein Labeling Kit RED-NHS (NanoTemper technologies) according to the manufacturer’s instructions. Labeled FcγR1 and FcγR2B were then coincubated with a 1:2 dilution series (12 steps) of human intravenous immunoglobulins (IVIGs, Privigen) starting at 50 mg/ml. For binding analyses the mixture was absorbed into Monolith NT.115 MST Premium Coated Capillaries (NanoTemper technologies) and FcγR binding to human IgG was analyzed on the Monolith NT.115 instrument (NanoTemper technologies) using the sequence 5 sec baseline, 30 sec IR laser on, 5 sec IR laser off. The normalized mean fluorescence signal of 4 measurements was plotted against the concentration of unlabeled IVIGs.

### Recombinant expression of FcγRs and ARTC2.1 in HEK cells

Codon-optimized open reading frames encoding for mouse FcγR1 (UniProtKB P26151) and FcγR2B (NCBI Reference Sequence: NP_001070657.1), flanked by EcoRI and NotI restriction sites were synthesized by GeneArt (Invitrogen) and cloned into the pCMVSport6.1 vector. Expression constructs were transfected into human embryonic kidney (HEK) cells using jetPEI transfection reagent (Q-Biogen). FcγR expression constructs were either transfected alone or co-transfected with pCMVSport6.1 encoding for ARTC2.1 kindly provided by Prof. Koch-Nolte.

### Phagocytosis assay

Adherent mixed glial cells from WT and ARTC2.1^−/−^ mice were stimulated with LPS/U0126 or IFNβ to induce ecto-ART activity in microglia, or left unstimulated. Cells were then pre-incubated with 100 µM NAD^+^/2 mM DTT or 2 mM DTT alone in 24-well plates for 30 min on ice. Cells were washed twice with 500 µl PBS to remove the NAD^+^/DTT and were incubated with PE-labeled IgG-coated latex beads (Cayman Chemical) at a dilution of 1:200 in BME 10% FCS medium at 37 °C. Mixed glial cells were allowed to phagocytose for 3 h, then the phagocytic activity of microglia was evaluated by flow cytometry. For the analyses, phagocytic activity was normalized to controls that were pre-incubated only with DTT (100%).

## Electronic supplementary material

Supplementary Data
