# Supplementary material for: Ecto-ADP-ribosyltransferase ARTC2.1 functionally modulates FcγR1 and FcγR2B on murine microglia
Source: Sci Rep. 2017 Nov 28;7:16477. doi: 10.1038/s41598-017-16613-w (PMC5705771; doi:10.1038/s41598-017-16613-w)
Supplement: Supplementary file 1 — Supplementary Data [file 41598_2017_16613_MOESM1_ESM.pdf]

Ecto-ADP-ribosyltransferase ARTC2.1 functionally modulates FcγR1 and FcγR2B on murine microglia

Björn Rissiek[1], Stephan Menzel[2], Mario Leutert[3], Maike Cordes[1], Sarah Behr[1], Larissa Jank[1], Peter Ludewig[1], Mathias Gelderblom[1], Anne Rissiek[2], Sahil Adriouch[2,4], Friedrich Haag[2], Michael O. Hottiger[3], Friedrich Koch-Nolte[2†], Tim Magnus[1†]

[1]Department of Neurology and [2]Institute of Immunology at
University Medical Centre, Hamburg-Eppendorf, Germany;
[3]Department of Molecular Mechanisms of Disease, University of Zurich, Switzerland
[4]Normandie Univ, UNIROUEN, INSERM, U1234, Rouen, France; Institute for Research and Innovation in Biomedicine (IRIB), Rouen, France.

Equally contributing †senior authors

To whom correspondence should be addressed:

Dr. Björn Rissiek
University Medical Center Hamburg-Eppendorf
Department of Neurology
Martinistraße 52
20246 Hamburg-Eppendorf
Germany
Tel.: +49-40-7410-58168
Fax.: +49-40-7410-55591
E-mail: b.rissiek@uke.de

Supplementary Information

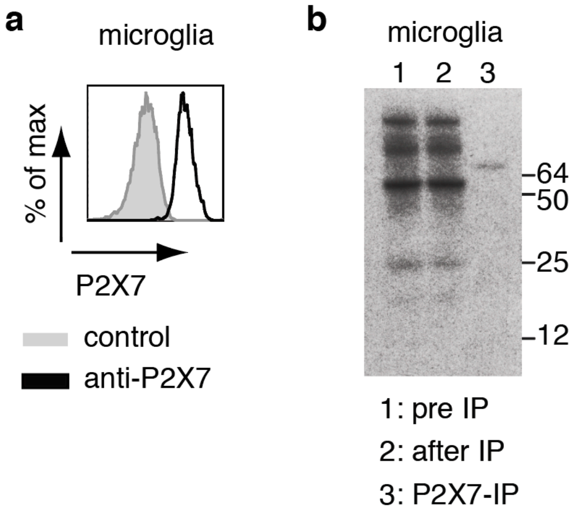

Supplementary Figure 1: ADP-ribosylation of P2X7 on microglia

(a) $CD11b^{+}$ microglia from BALB/c WT mixed glial cells were labeled with anti-P2X7 or isotype control antibodies. **(b)** BALB/c WT mixed glial cells were stimulated with LPS/U0126 for 24h and microglia were isolated via FACS sorting of $CD11b^{+}GLAST^{-}$ cells. Sorted cells were lysed and P2X7 protein was immunoprecipitated (IP) using anti-P2X7 (RH23A44). Pre-IP, after-IP and P2X7-IP samples were size fractionated on SDS PAGE and subjected to autoradiography. Data are representative of 2 independent experiments.
